# Notes on molecular fragmentation and parameter settings for a dissipative particle dynamics study of a C_10_E_4_/water mixture with lamellar bilayer formation

**DOI:** 10.1186/s13321-023-00697-w

**Published:** 2023-02-19

**Authors:** Felix Bänsch, Christoph Steinbeck, Achim Zielesny

**Affiliations:** 1 Institute for Bioinformatics and Chemoinformatics, Westphalian University of Applied Sciences, August-Schmidt-Ring 10, 45665 Recklinghausen, Germany; 2 Institute for Analytical Chemistry, Friedrich-Schiller-University, Jena, Germany

**Keywords:** Dissipative particle dynamics, DPD, Surfactant, Bilayer, Lamellar, Simulation, Mesoscopic

## Abstract

The influence of molecular fragmentation and parameter settings on a mesoscopic dissipative particle dynamics (DPD) simulation of lamellar bilayer formation for a C_10_E_4_/water mixture is studied. A “bottom-up” decomposition of C_10_E_4_ into the smallest fragment molecules (particles) that satisfy chemical intuition leads to convincing simulation results which agree with experimental findings for bilayer formation and thickness. For integration of the equations of motion Shardlow’s S1 scheme proves to be a favorable choice with best overall performance. Increasing the integration time steps above the common setting of 0.04 DPD units leads to increasingly unphysical temperature drifts, but also to increasingly rapid formation of bilayer superstructures without significantly distorted particle distributions up to an integration time step of 0.12. A scaling of the mutual particle–particle repulsions that guide the dynamics has negligible influence within a considerable range of values but exhibits apparent lower thresholds beyond which a simulation fails. Repulsion parameter scaling and molecular particle decomposition show a mutual dependence. For mapping of concentrations to molecule numbers in the simulation box particle volume scaling should be taken into account. A repulsion parameter morphing investigation suggests to not overstretch repulsion parameter accuracy considerations.

## Introduction

Dissipative particle dynamics (DPD) is a mesoscopic simulation technique for isothermal complex fluids and soft matter systems. It satisfies Galilean invariance and isotropy, conserves mass and momentum and achieves a rigorous sampling of the canonical NVT ensemble due to soft particle pair potentials that diminish molecular entanglements or caging effects. DPD is expected to show correct hydrodynamic behavior and to obey the Navier–Stokes equations [1–7]. DPD particle trajectories are guided by Newton’s equation of motion,

$$m_{i} \frac{{d^{2} \underline{r}_{i} }}{{dt^{2} }} = \underline{F}_{i} = \sum\limits_{\begin{subarray}{l} j = 1 \\ j \ne i \end{subarray} }^{N} {\left( {\underline{F}_{ij}^{C} + \underline{F}_{ij}^{D} + \underline{F}_{ij}^{R} } \right)}$$

*with *$m_{i} ,\underline{r}_{i}$*, mass and position vector of particle i; *$t$*, time; *$\underline{F}_{i}$*, total force on particle i exerted by other particles j; *$N$*, number of particles in simulation; *$\underline{F}_{ij}^{C} ,\underline{F}_{ij}^{D} ,\underline{F}_{ij}^{R}$*, conservative, dissipative and random force on particle i exerted by particle j.*.

Dissipative (frictional) force

$$\underline{F}_{ij}^{D} \left( {\underline{r}_{ij} ,\underline{v}_{ij} } \right) = - \gamma \;\omega^{D} \left( {r_{ij} } \right)\;\left( {\underline{r}_{ij}^{0} \cdot \underline{v}_{ij} } \right)\;\underline{r}_{ij}^{0}$$

*with *$\gamma$*, friction coefficient; *$\omega^{D} \left( {r_{ij} } \right)$*, dissipative force distance variation; *$\underline{v}_{i}$*, velocity of particle i; *$\underline{v}_{ij} = \underline{v}_{i} - \underline{v}_{j}$*;*

and random force,

$$\underline{F}_{ij}^{R} \left( {\underline{r}_{ij} } \right) = \sigma \;\omega^{R} \left( {r_{ij} } \right)\;\,\frac{{\zeta_{ij} }}{{\sqrt {\Delta t} }}\;\underline{r}_{ij}^{0}$$

*with *$\sigma$*, noise amplitude; *$\omega^{R} \left( {r_{ij} } \right)$*, random force distance variation; *$\,\zeta_{ij}$*, random number with zero mean and unit variance; *$\Delta t$*, integration time step;*

oppose each other with mutual dependence (where a common cut-off length of 1 DPD unit is applied),

$$\begin{gathered} \gamma = \frac{{\sigma^{2} }}{{2\;k_{B} T}} \\ \omega^{R} \left( {r_{ij} } \right) = \sqrt {\omega^{D} \left( {r_{ij} } \right)} = \left\{ {\begin{array}{*{20}c} {1 - r_{ij} } & {{\text{ for }}r_{ij} < 1} \\ 0 & {{\text{ for }}r_{ij} \ge 1} \\ \end{array} } \right. \\ \end{gathered}$$

*with* $k_{B}$,* Boltzmann constant; *$T$*, thermodynamic temperature.* and act as a thermostat conserving the total momentum and introducing Brownian motion into the system. The conservative forces comprise soft DPD particle repulsions (again with a common cut-off length of 1 DPD unit) and harmonic springs between bonded particles,

$$\begin{gathered} \underline{F}_{ij}^{C} = \underline{F}_{ij}^{C,DPD} + \underline{F}_{ij}^{C,Bond} \\ \underline{F}_{ij}^{C,DPD} \left( {\underline{r}_{ij} } \right) = \left\{ {\begin{array}{*{20}c} {a_{ij} \left( {1 - r_{ij} } \right)\underline{r}_{ij}^{0} } & {{\text{ for }}r_{ij} < 1} \\ 0 & {{\text{ for }}r_{ij} \ge 1} \\ \end{array} } \right. \\ \underline{F}_{ij}^{C,Bond} = - k_{Bond} \,\left( {r_{ij} - r_{Bond} } \right)\,\,\underline{r}_{ij}^{0} \\ \end{gathered}$$

*with *$\underline{F}_{ij}^{C,DPD} ,\underline{F}_{ij}^{C,Bond}$*, soft repulsive DPD force and harmonic bond force on particle i exerted by particle j; *$a_{ij}$*, maximum isotropic repulsion between particles i and j; *$\underline{r}_{ij} = \underline{r}_{i} - \underline{r}_{j} = r_{ij} \,\underline{r}_{ij}^{0}$; $\underline{r}_{ij}^{0}$, *unit vector; *$k_{Bond}$*, spring constant of bond; *$r_{Bond}$*, bond length.*

The isotropic repulsions $a_{ij}$ determine the particle–particle interactions and thus the dynamical behavior of a molecular ensemble under study. They may be derived from Flory–Huggins interaction parameters [5],

$$a_{ij} \left( T \right) = 75\frac{{k_{B} T}}{{\rho_{DPD} }} + 3.4965\;k_{B} T\;\chi_{ij} \left( T \right){ ; }\chi_{ii} = 0$$

*with *$\rho_{DPD}$*, DPD density; *$\chi_{ij}$*, Flory–Huggins interaction parameter between particles i and j; *$Z_{ij}$*, coordination number of particles j around particle i; *$E_{ij}$*, interaction energy between particles i and j.*

Each DPD simulation study requires deliberate choices for adequate molecular particle decompositions, the isotropic particle–particle repulsions $a_{ij}$ as well as the physical parameter settings that guide the simulation. These choices are commonly derived from theoretical considerations, experimental findings, experience or a set of educated guesses and are often contentious issues concerning the scientific validity of a particular simulation setup. In this work an alternative approach is followed which is based on a specific molecular ensemble (a binary non-ionic surfactant/water mixture) with an experimentally well-characterized (lamellar bilayer) superstructure: Since the correct simulation result is known in advance, different molecular particle decompositions and settings (integration type, integration time step, particle–particle repulsion scaling, particle volume scaling, repulsion parameter morphing) can be assessed for success or failure and general advice for their interplay may be obtained.

## Methods

**Fig. 1:**
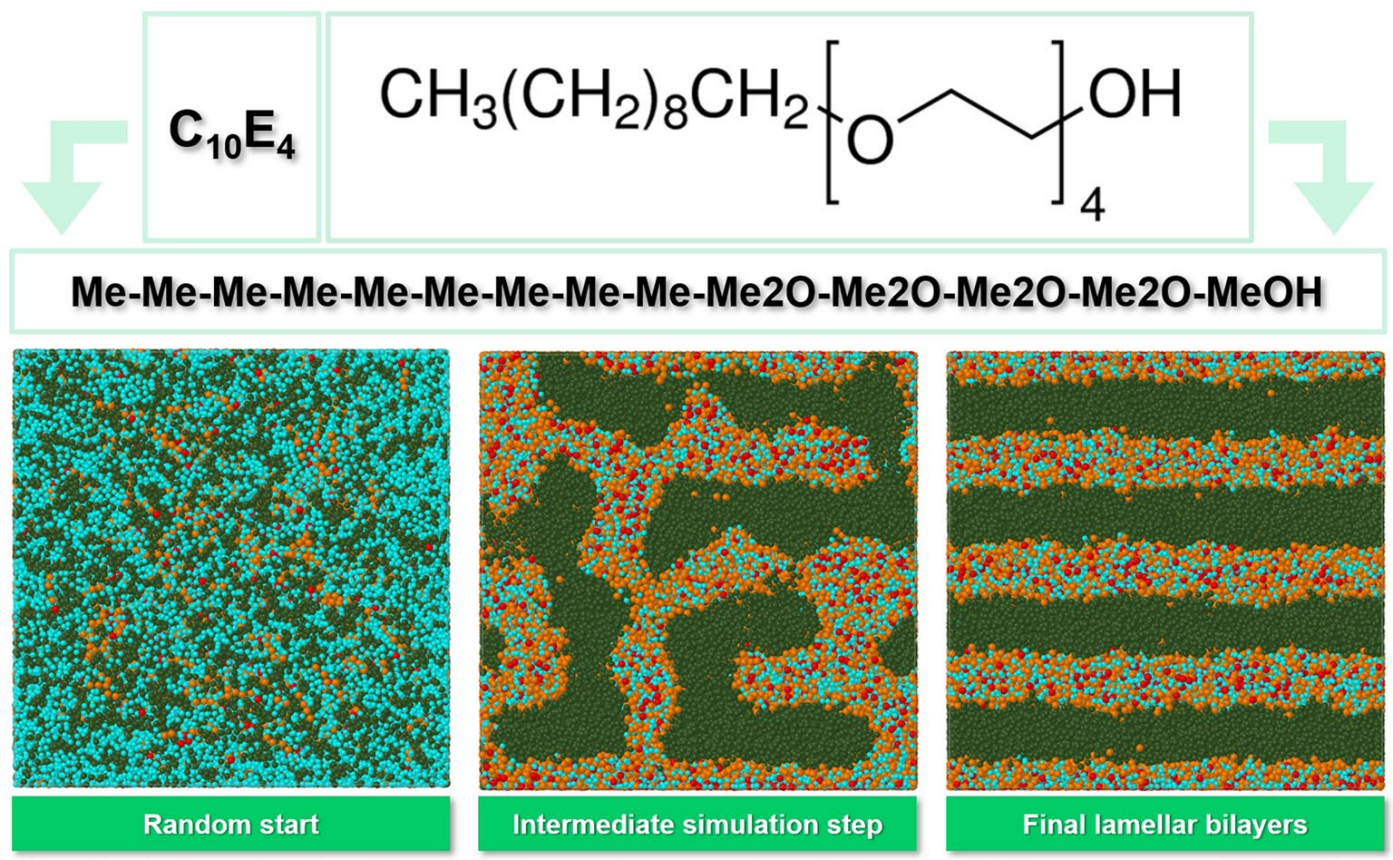
Top: Molecular fragmentation of non-ionic surfactant C_10_E_4_ into SPICES string 9Me-4Me2O-MeOH (with particles Me: Methane, Me2O: Dimethylether, MeOH: Methanol) and topological depiction of the particle bonds. Bottom: Front (horizontal x-, vertical z-axis) cross-section view of the simulation box from random start geometry over an intermediate simulation step up to the final lamellar bilayer structure perpendicular to the vertical z-axis (particle colors: Me: Olive, Me2O: Orange, MeOH: Red, H2O: Cyan)

The specific molecular ensemble is chosen to be a binary mixture of water with the non-ionic polyoxyethylene alkyl ether surfactant 3,6,9,12-tetraoxadocosan-1-ol, CH_3_(CH_2_)_8_CH_2_(OCH_2_CH_2_)_4_OH, abbreviated C_10_E_4_ where “C_10_” indicates the number of carbon atoms in the alkyl chain of the lyophobic part and “E_4_” represents the number of lyophilic ethylene oxide units, see top of Fig. 1.

Lang and Morgan [8] and Stubenrauch et al. [9] have shown by different characterization techniques that a C_10_E_4_/water mixture forms a stable lamellar L_α_ phase around 298 K for a C_10_E_4_ mass fraction of 0.75. Lang and Morgan identified the lamellar phase by X-ray powder diffraction and measured the C_10_E_4_ mass fraction gravimetrically. Stubenrauch et al. utilized light scattering and ^2^H-NMR spectroscopy to determine the phase behavior.

Starting from a random mixture of C_10_E_4_ and water molecules, the formation of the lamellar C_10_E_4_ bilayer structure on the microsecond scale can be studied using the mesoscopic DPD simulation technique (see bottom of Fig. 1 and the simulation clip at [10]). A suitable molecular particle decomposition for C_10_E_4_ (SPICES string 9Me-4Me2O-MeOH [11, 12], see Fig. 1) and adequate estimates for the corresponding particle pair repulsion parameters $a_{ij}$ are taken from [13]. A water molecule is represented by a single DPD particle/bead, which is the smallest particle of a particle set with a volume of 30 Å^3^.

**Fig. 2:**
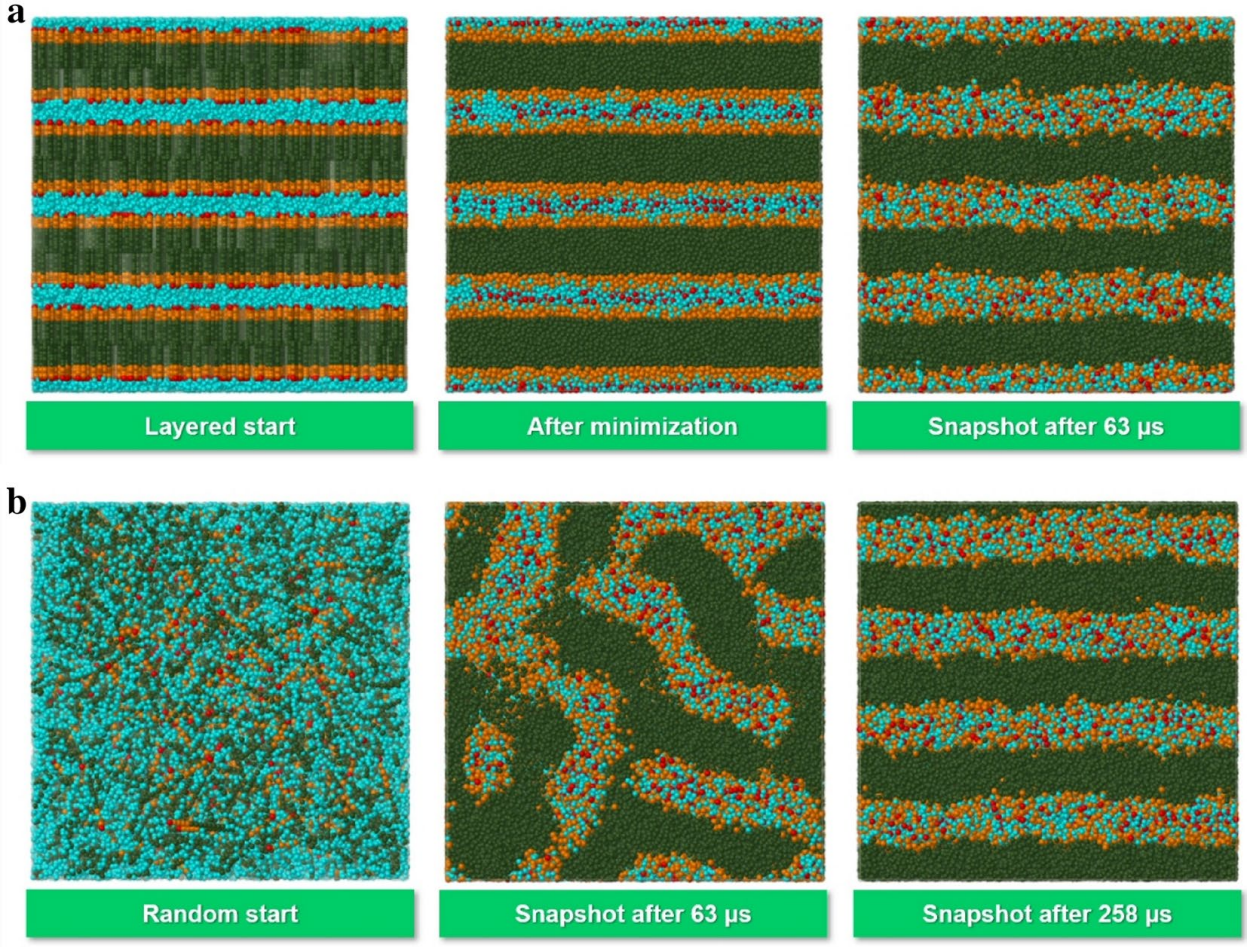
Optimal (minimum energy) superstructure formation of a binary C_10_E_4_/water mixture at 298 K with a C_10_E_4_ mass fraction of 0.75 with periodic boundary conditions in all directions and an integration time step of 0.04. Top row: Front (horizontal x-, vertical z-axis) cross-section view of the simulation box for a layered start geometry (left), minimized start geometry (middle) and simulation snapshot after one million simulation steps (63 µs, right). Bottom row: Front (horizontal x-, vertical z-axis) cross-section view of the simulation box for a random start geometry (left), simulation snapshot after one million simulation steps (63 µs, middle) and simulation snapshot after 4,112 million simulation steps (258 µs, right) with completed formation of stacked bilayers. For the same random start geometry with disabled periodic boundary in z-direction the stacked bilayer superstructure emerges after 74 thousand simulation time steps (4 µs), i.e. more than 60 times faster. The particle colors are identical to those in Fig. 1

In a direct manner, the optimal stacked C_10_E_4_ bilayer superstructure results from a suitable layered start geometry in which the bilayers are arranged perpendicular to the z-axis of the simulation box (see top row of Fig. 2): After minimizing the energy of the initial start geometry with a number of adequate “force steps”, this minimum energy superstructure is conserved during simulation using periodic boundaries in all directions. To arrive at this optimal superstructure (not necessarily in the xy-plane) from initial random mixing of C_10_E_4_ and water molecules (see bottom row of Fig. 2), millions of simulation steps may be required since the energy gradient for the superstructure formation is very small. This would lead to unacceptably long times for a single simulation run in a comparative study with a considerable number of jobs. Therefore, periodic boundaries in z-direction are disabled, which significantly accelerates the formation of C_10_E_4_ bilayers parallel to the xy-plane by introducing a preferred direction that appropriately shapes the structure formation to its true optimum, so that convergence can be achieved within hundreds of thousands of simulation steps. The definition of reflective walls in z-direction inevitably leads to artefacts in the particle distribution compared to the optimal superstructure with periodic boundaries in all directions (e.g., the particle density oscillations near the walls clearly seen in Fig. 4 below), but these do not affect the investigations of this work.

For investigation of the effect of the relative magnitude of the $a_{ij}$ parameters, the off-diagonal Flory–Huggins interaction parameter $\chi_{ij}$ contributions are linearly scaled to different ranges: There are three corresponding particles sets constructed with “range 10” [14], “range 15” [15] and “range 20” [16], where “range x” denotes the maximum absolute deviation of “x” between the smallest $a_{ij}$ value and the diagonal value $a_{ii} = 24.83$ for a thermodynamic temperature of 298 K.

In addition to the off-diagonal $a_{ij}$ scaling effects, the influence of $a_{ij}$ morphing is studied by shifting the repulsions of the Dimethylether (Me2O) particle towards the repulsions of the (most hydrophilic) methanol (MeOH) particle so that they finally exhibit equal repulsions. The degree of the shift may be characterized by a percentage of the absolute $a_{ij}$ difference between the two particles in question with a third particle where the shift for the repulsion between the two particles in question runs against the diagonal value $a_{ii}$.

The performance of different integration types with different integration time steps is evaluated—in particular the original Groot-Warren scheme (GWMVV, MVV: Modified Velocity-Verlet) [5, 17, 18] which depends on a tuning parameter where GWMVV equals Velocity-Verlet (VV) integration for a value of 0.5, the self-consistent scheme (SCMVV) [17–19] with an adjustable number of self-consistent dissipative force iterations where a single iteration leads to the DPDMVV variant, Shardlow’s S1 scheme (S1MVV) [20, 21] and the Nonsymmetric Pairwise Noose-Hoover-Langevin thermostat (PNHLN) [22] that requires the definition of an additional coupling parameter.

To study the influence of different molecular fragmentation schemes, the known adequate C_10_E_4_ fragmentation with SPICES string 9Me-4Me2O-MeOH (denoted scheme A) from [13] is changed to 4Et-Me-4Me2O-MeOH (scheme B) where two methane (Me) particles are replaced by a corresponding ethane (Et) particle.

The conversion between DPD lengths and physical lengths is based on the conversion radius $r_{c}$ (“radius of interaction”) in physical units

$$r_{c} = \sqrt[3]{{V_{\min } \;\rho_{DPD} \frac{{\sum\limits_{i = 1}^{{N_{particles} }} {N_{particle,i} \frac{{V_{particle,i} }}{{V_{\min } }}} }}{{\sum\limits_{i = 1}^{{N_{particles} }} {N_{particle,i} } }}}}{ ; }\,l_{phys} = l_{DPD} \;r_{c}$$

*with *$V_{\min }$*, volume of smallest particle in physical units; *$\rho_{DPD}$*, DPD (number) density; *$N_{particles}$*, number of different particle types; *$N_{particle,i}$*, number of particles of type i; *$V_{particle,i}$*, volume of particle of type i in physical units; *$l_{phys}$*, length in physical units; *$l_{DPD}$*, length in DPD units;*

with the conversion between DPD and physical time being approximated by

$$t_{phys} = t_{DPD} \;f_{soft} \;r_{c} \;\sqrt {\frac{1}{R\;T}\frac{{\sum\limits_{i = 1}^{{N_{particles} }} {N_{particle,i} \;M_{particle,i} } }}{{\sum\limits_{i = 1}^{{N_{particles} }} {N_{particle,i} } }}}$$

*with *$t_{phys}$*, time in physical units; *$t_{DPD}$*, time in DPD units; *$f_{soft} \approx 1000$*, factor for increased particle diffusivity due to soft potentials; *$R$*, gas constant; *$T$*, thermodynamic temperature; *$M_{particle,i}$*, molar mass of particle of type i.*

The need for volume scaling in concentration calculations is analyzed, with the conversion of the binary mixture composition to the specific volume-scaled molecular numbers of the simulation box described in Appendix 1 of [23]. For 18,500 C_10_E_4_ molecules and a C_10_E_4_ mass fraction of 0.75 this leads to 79,174 water molecules for C_10_E_4_ fragmentation scheme A and 65,337 water molecules for scheme B.

Common parameter settings for DPD simulations are used: $\rho_{DPD} = 3, \, \sigma = 3, \, k_{Bond} = 4, \, r_{Bond} = 1$, mass of particle i $m_{particle,i} = 1$. Particle volumes are only used for physical length/time related conversions from DPD units (see above).

All DPD simulations of this study are performed with the open simulation environment MFsim [23, 24] using the Jdpd simulation kernel [25, 26]. The maximum simulation period chosen comprised one million simulation steps which corresponds to a physical time span in the order of 10–100 microseconds depending on the integration time step. A single simulation job run is finished within 20–40 h (depending on the defined integration scheme) using 8 parallelized Jdpd calculation threads on a modest multi-core workstation. The MFsim simulation jobs are openly documented at [27]. All simulations of this work were repeated several times with different settings of the seed value for random number generation (MFsim/Jdpd parameter *Geometry random seed* in the *JobInput/Chemical system description/Simulation box* section for job definition) so that different random start geometries were created. The reported values correspond to single simulation runs with a representative (intermediate) result. Any significant deviations due to different initial random particle positions are pointed out in the “Results” section.

## Results

### S1MVV integration with C_10_E_4_ fragmentation scheme A

**Fig. 3:**
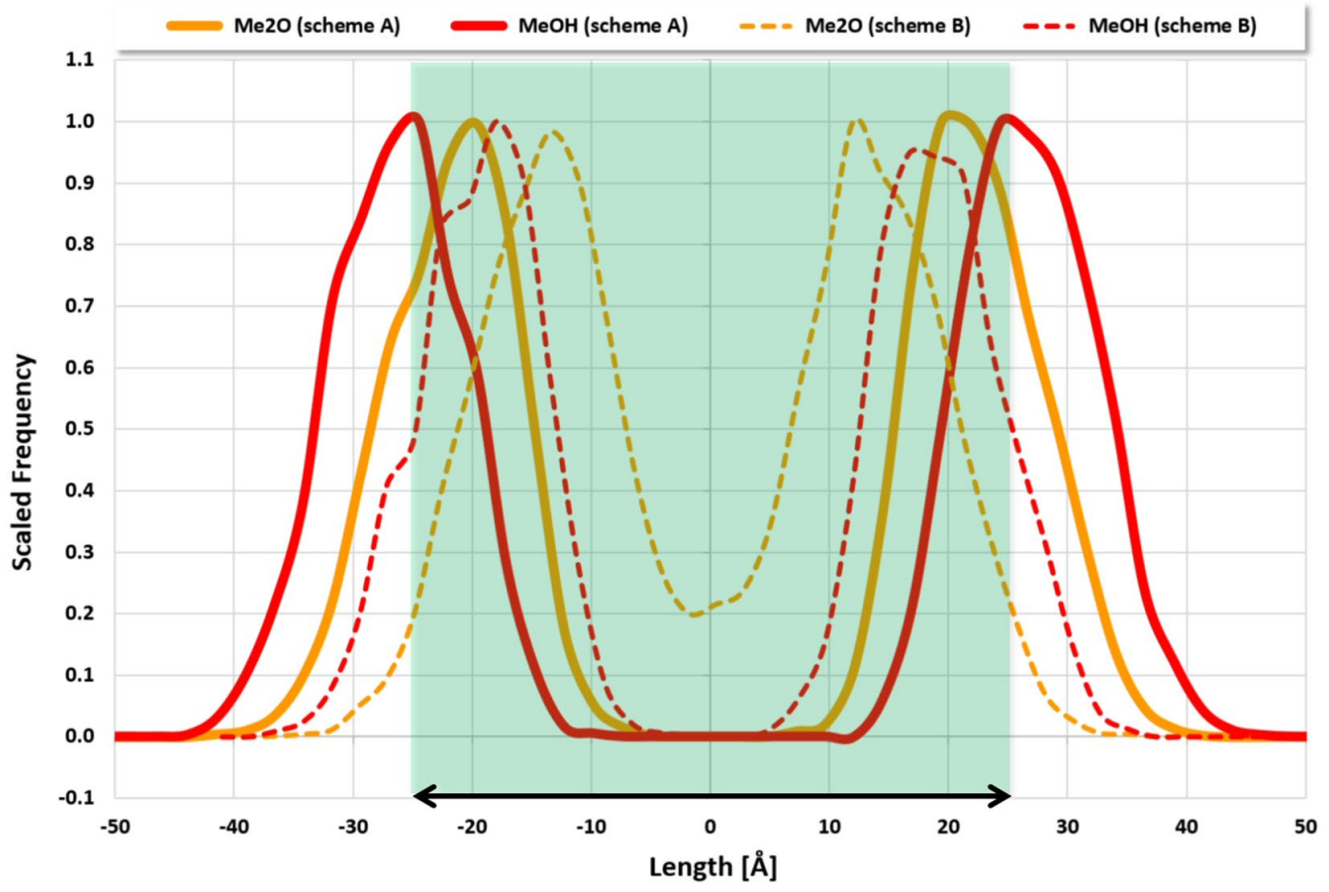
Me2O and MeOH particle distribution snapshot along the z-axis perpendicular to a single C_10_E_4_ bilayer (S1MVV integration with time step of 0.01, “range 20” particle set, step one million). The highlighted area in light green corresponds to a width of 50 Å as indicated by the double arrow. Thick solid lines: C_10_E_4_ fragmentation scheme A, thin dashed lines: C_10_E_4_ fragmentation scheme B

Simulating the C_10_E_4_/water mixture with an initial random start geometry for one million simulation steps using C_10_E_4_ fragmentation scheme A, S1MVV integration with a time step of 0.01 and the “range 20” particle set leads to a perfect lamellar bilayer structure at step 414,000 (physical time of about 7 µs) as a stable superstructure, see simulation clips at [10] and [28]. For different initial random start geometries, this convergence varies on the order of tens of thousands of time steps (less than 0.5 µs in physical time). The emerged C_10_E_4_ bilayers exhibit thicknesses that are in good agreement with the value of 50 Å estimated on the basis of neutron reflectivity measurements [9], see Fig. 3. The temperature remains stable at 298.2 K throughout the whole simulation with a small positive temperature drift of 0.2 K compared to its initial setting of 298.0 K. The DPD surface tension in z-direction is sensitive to C_10_E_4_ bilayer formation perpendicular to the z-axis and increases until a plateau with stable C_10_E_4_ bilayers is reached.

**Fig. 4:**
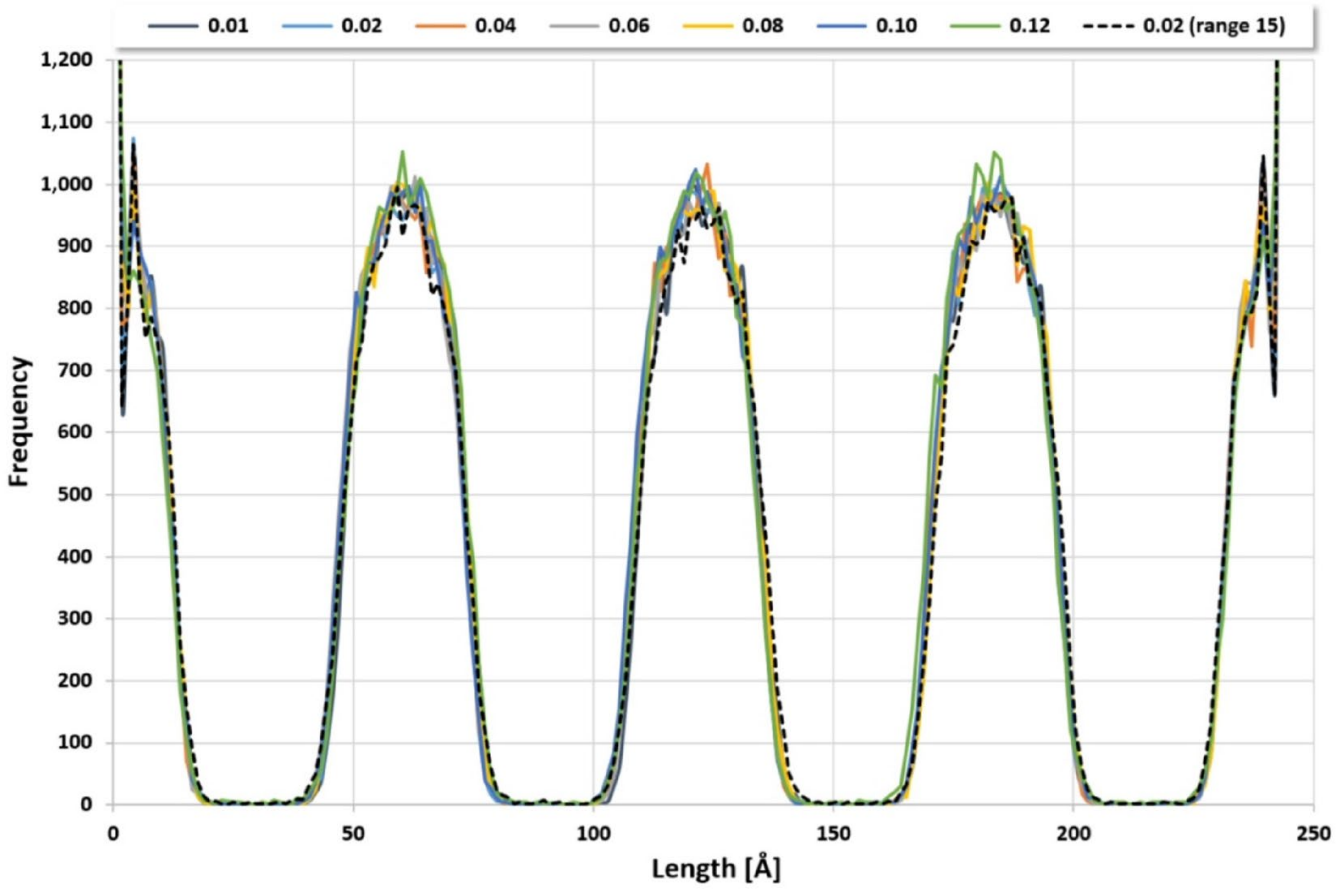
Distribution of water particles in the simulation box along the z-axis for different integration time steps that lead to a C_10_E_4_ bilayer superstructure formation (C_10_E_4_ fragmentation scheme A, S1MVV integration, snapshot of last simulation step one million). The integration time steps are reported in the legend above and refer to the “range 20” particle set with exception of the 0.02 integration time step of “range 15” particle set (dashed black line)

A doubled integration time step of 0.02 leads to the same qualitative findings but roughly halved 220,000 simulation steps for C_10_E_4_ bilayer superstructure formation with a positive temperature drift being increased to 0.8 K. A change of the seed for random number generation reproduces the sketched findings but leads to different formation times of the C_10_E_4_ bilayer superstructure: For four different seed values for initial random position generation the number of necessary formation steps changes from 220,000 steps to values between 190,000 and 244,000 steps. A further increase of the integration time step decreases the necessary number of simulation steps for C_10_E_4_ bilayer superstructure formation with an increasingly positive temperature drift (see Table 1). An integration time step greater than 0.12 prohibits a C_10_E_4_ bilayer superstructure formation and keeps a random mixture. The resulting particle distributions in the simulation box for all integration time steps with C_10_E_4_ bilayer superstructure formation are nearly congruent, see Fig. 4.

**Table 1:** “Range 20” particle set

Integration time step [DPD]	0.01	0.02	0.04	0.06	0.08	0.10	0.12
Convergence [in 1000 simulation steps]	414	220	84	54	34	20	14
Temperature drift [K]	0.2	0.8	3.2	8.0	17.2	39.2	141.4

If the previous simulations (C_10_E_4_ with fragmentation scheme A, S1MVV integration) are repeated with the “range 15 “ particle set instead of the “range 20” particle set the reported results remain basically unchanged, compare Table 2 and Fig. 4: The particle distributions are similar, the convergence towards the bilayer superstructure is on average slightly slower with slightly increased temperature drifts. Again, an integration time step greater than 0.12 prohibits a C_10_E_4_ bilayer superstructure formation and keeps a random mixture.

**Table 2:** “Range 15” particle set

Integration time step [DPD]	0.01	0.02	0.04	0.06	0.08	0.10	0.12
Convergence [in 1000 simulation steps]	464	232	106	64	52	36	18
Temperature drift [K]	0.2	0.9	3.5	8.5	18.2	41.0	146.9

A corresponding repetition of the simulations with the “range 10” particle set no longer allows for a distinct C_10_E_4_ bilayer superstructure formation within one million simulation steps regardless of the integration time step.

### Other integration types with C_10_E_4_ fragmentation scheme A

The original GWMVV/0.65 integration (with a tuning parameter of 0.65 [5]) performs slightly (10%) faster than the S1MVV scheme [25]. With the “range 15” particle set the GWMVV/0.65 integration leads to results comparable with S1MVV integration for the smallest integration time steps of 0.01, 0.02 and 0.04 (see Table 3) and particle distributions shown in Fig. 4, where the potential DPD and bond energies of the C_10_E_4_ bilayer plateau region are above the values of S1MVV integration. For integration time steps of 0.06 or higher GWMVV/0.65 integration does no longer converge to distinct C_10_E_4_ bilayers but to twisted (0.06) and bridged (0.08) layer structures or random mixtures for integration time steps of 0.10 or higher. For the “range 10” particle set GWMVV/0.65 integration also no longer allows for a distinct C_10_E_4_ bilayer superstructure formation within one million simulation steps.

**Table 3:** GWMVV/0.65 integration and “range 15” particle set

Integration time step [DPD]	0.01	0.02	0.04
Convergence [in 1000 simulation steps]	508	300	166
Temperature drift [K]	0.7	1.2	2.7

Compared to the S1MVV scheme, DPDMVV integration (with one self-consistent dissipative force iteration, i.e. SCMVV/1) is slightly (10%) slower whereas SCMVV/5 integration (with five self-consistent dissipative force iterations) is significantly slower by a factor of 2. With the “range 15” particle set both integration types lead to results which are similar to those of S1MVV integration. For DPDMVV the positive temperature drift is slightly increased, for SCMVV/5 the temperature drift also exhibits negative values and is reduced in magnitude even slightly below S1MVV integration. The potential DPD and bond energies of the C_10_E_4_ bilayer plateau region are enhanced for DPDMVV but equal to those of the S1MVV scheme for SCMVV/5. The particle distributions are similar and for integration time steps greater 0.12 only random mixtures are generated alike the findings for S1MVV.

The PNHLN integration scheme roughly doubles the necessary computational time span compared with GWMVV/0.65 or S1MVV integration and compares to SCMVV/5 [25]. For an (empirically evaluated adequate) coupling parameter of 500 and the “range 15” particle set the findings are in agreement with those of S1MVV integration for the smallest integration time steps of 0.01, 0.02 and 0.04 where PNHLN/500 integration exhibits very small positive temperature drifts of 0.1–0.2 K as well as potential DPD and bond energies of the C_10_E_4_ bilayer plateau region which are the smallest of all integration types studied. For integration time steps of 0.06 and higher the comparatively small temperature drifts remain but after the C_10_E_4_ bilayer superstructure is reached there are unexpected sudden intermediate “bilayer and water bridges” formed that correspond to jumps of the potential DPD and bond energies. An integration time step of 0.14 or higher fails since particles “are thrown out of the simulation box” (note that Jdpd provides safeguards against this unwanted behavior—but nonetheless this should “never happen”). There is also a distinct anomaly in the progress of the potential DPD energy for integration time steps of 0.04 or higher: It decreases until the C_10_E_4_ bilayer superstructure is formed but then starts to rise again. This finding is the more pronounced the larger the integration time step is defined. For the “range 20” particle set the anomaly does not occur for an integration time step of 0.04 but emerges for a length of 0.06 or higher, the unexpected sudden intermediate “bilayer and water bridges” with jumps of the potential DPD and bond energies do only occur for integration time step of 0.10 and 0.12. It should be noted that the sketched behavior may not be removed by altering the PNHLN coupling parameter value. Like GWMVV and S1MVV integration there is no distinct C_10_E_4_ bilayer superstructure formation within one million simulation steps for the “range 10” particle set.

### S1MVV integration with C_10_E_4_ fragmentation scheme B

C_10_E_4_ fragmentation scheme B with SPICES string 4Et-Me-4Me2O-MeOH (denoted scheme A) replaces two methane (Me) particles by an ethane (Et) particle. A S1MVV integration with a time step of 0.01 and the “range 20” particle set again leads to a perfect lamellar bilayer structure which is completely emerged at step 534,000 (physical time of about 10 µs) as a stable superstructure. The C_10_E_4_ bilayers exhibit reduced thicknesses of only 35 Å which are well below the findings for fragmentation scheme A and experiment, see Fig. 3 (the reduced C_10_E_4_ bilayer thicknesses now allow for five bilayers in the simulation box instead of four bilayers for fragmentation scheme A, compare Figs. 1 and 4). Corresponding simulations with the GWMVV/0.65 and the PNHLN/500 integration instead of S1MVV lead to equal results (all with a comparable small positive temperature drift as found for the scheme A simulations). Using the “range 15” instead of the “range 20” particle set leads to faster convergence with comparable results. In contrast to all scheme A simulations the “range 10” particle set also allows for a comparable convergence to the stable bilayer structure for the integration types studied.

### Influence of volume scaling for concentration calculations

**Fig. 5:**
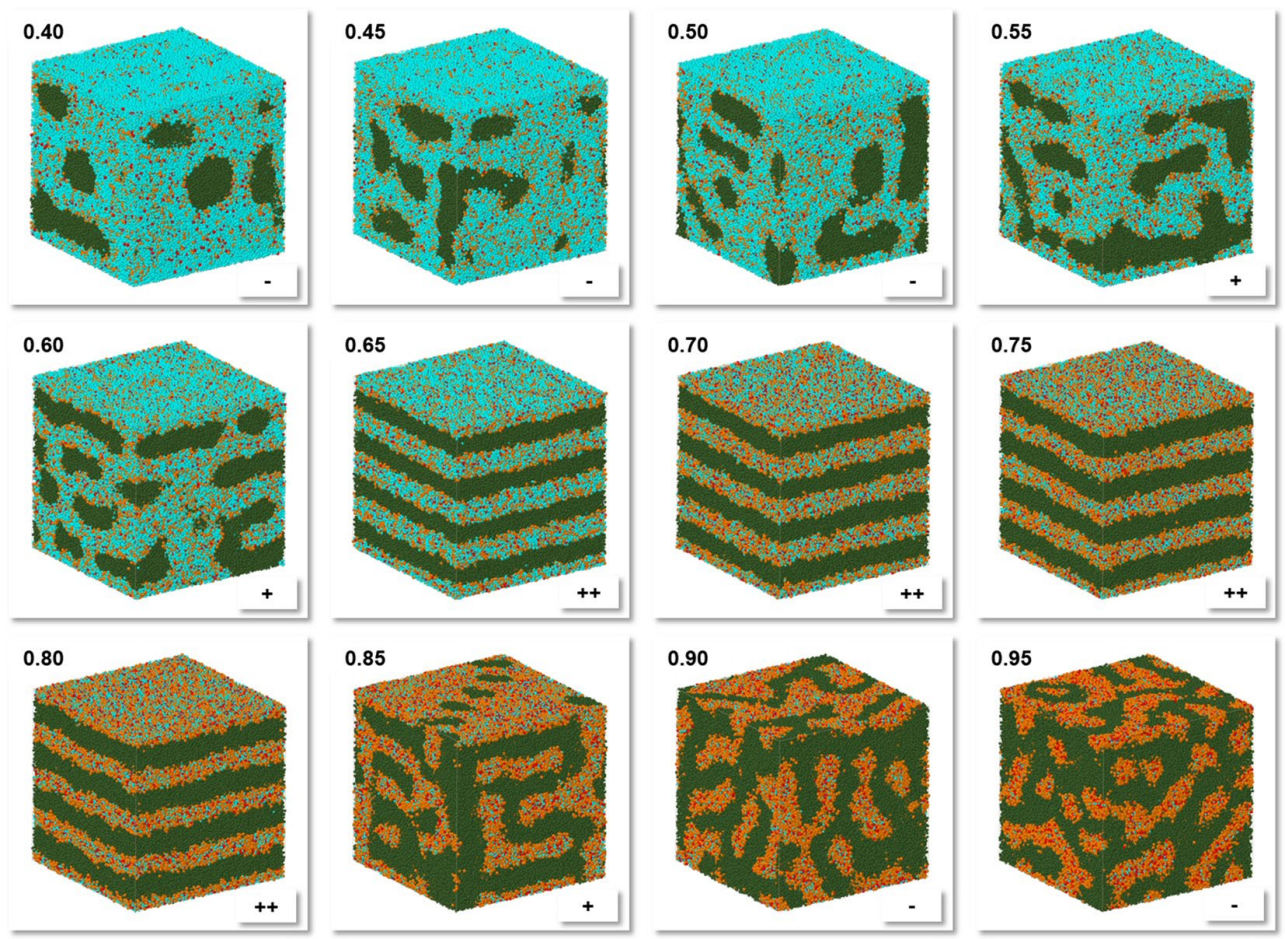
Final particle/molecule distributions for different volume-scaled mass fractions (upper left corner) of C_10_E_4_ at 298 K (with C_10_E_4_ fragmentation scheme A, S1MVV integration, integration time step 0.04, snapshot of last simulation step 500,000 which corresponds to 30 microseconds) with particle colors: Me: Olive, Me2O: Orange, MeOH: Red, H2O: Cyan. Lower right corner: Change of DPD surface tension in z-direction (perpendicular to the C_10_E_4_ bilayers) during simulation from random start geometry where “−”: No change, “+”: Positive rise smaller 6 DPD units, “+ +”: Positive rise larger 7 DPD units

The use of volume scaling for the conversion of mixture compositions to corresponding particles numbers in the simulation box leads to decreased numbers of particles with smaller volumes. Thus, for a C_10_E_4_/water mixture volume scaling reduces the number of the (smallest) water particles. The C_10_E_4_/water system allows to assess this common technique by simulation of mixtures with different compositions at 298 K. The C_10_E_4_/water phase diagram [8, 9] shows that the lamellar L_α_ phase at the 298 K isotherm is enclosed by two isotropic phases and spans a C_10_E_4_ mass fraction from about 0.55 up to about 0.80. The corresponding simulation results with volume-scaled mass fractions depicted in Fig. 5 agree with these experimental findings where the rise of DPD surface tension in z-direction during simulation from the random start geometry is utilized as a sensitive measure for C_10_E_4_ bilayer formation perpendicular to the z-axis.

### Influence of repulsion parameter morphing

For the C_10_E_4_/water system a morphing procedure of the dimethylether (Me2O) particle towards the most hydrophilic methanol (MeOH) particle was carried out by construction of five morphing particle sets with shifts of 20%, 40%, 60%, 80% and 100% where the latter has equal $a_{ij}$ repulsions for Me2O and MeOH. The morphed “range 20” particle sets are available at [29]. Simulating the C_10_E_4_/water mixture with an initial random start geometry for one million simulation steps using C_10_E_4_ fragmentation scheme A and S1MVV integration with a time step of 0.01 the 20% and 40% shift particle sets lead to the same perfect lamellar bilayer structure with equal particle distributions as the non-shifted particle set but distinctly slower convergence: Whereas the non-shifted particle set leads to a perfect lamellar bilayer structure at step 414,000 (compare above) the 20% shift particle set requires 588,000 steps and the 40% shift particle set 686,000 steps. The 60% and higher shifted particle sets do no longer exhibit a convergence towards the lamellar bilayer structure perpendicular to the z-axis of the simulation box.

## Discussion and conclusions

A DPD simulation requires an initial choice of suitable particles with adequate interactions to properly describe the structure and dynamics of the system under study. Since there is no general objective particle decomposition framework to guide this choice, the particle partitioning procedure may at best follow plausible considerations within the specific field of application. For the C_10_E_4_/water mixture an approach is chosen which defines the water molecule as the smallest volume particle and partitions C_10_E_4_ into the smallest chemically adequate fragment molecules: Thus, the alkyl chain is partitioned into methane particles, the oxygen environment of the oxyethylene units is represented by dimethylether particles and the terminal hydroxyl group is abstracted with a methanol particle. The particle concatenation with harmonic springs reflects the linear surfactant geometry where the number of non-hydrogen atoms of the C_10_E_4_ molecule is conserved by the SPICES particle structure (Fig. 1). From chemical intuition, this fragmentation scheme A appears to be the basic “bottom-up” description of a C_10_E_4_ surfactant molecule. The final justification of particle choice and molecule fragmentation is of course their success for studying the system of interest, i.e. their ability to approximately describe elements of reality that this system exhibits. From a mesoscopic perspective, studying the C_10_E_4_/water mixture with C_10_E_4_ fragmentation scheme A, S1MVV integration with a small time step of 0.01 and the “range 20” particle set leads to a convincing DPD simulation result which satisfactorily agrees with qualitative and quantitative experimental findings as outlined above. In addition, the C_10_E_4_ fragmentation scheme A has been successfully applied to study equilibrium nanoscale structures at the water–air surface and to determine surface tensions [13].

An increase of the integration time step exhibits an expected acceleration of the C_10_E_4_ bilayer formation process with regard to the necessary number of integration steps, accompanied by increasing temperature drifts and higher potential plateau energies, until an upper time step threshold is reached that no longer allows for bilayer emergence. Interestingly, the increase of the integration time steps does not significantly affect the final particle distributions in the simulation box (see Figs. 3 and 4): Thus, from a pure particle distribution perspective, a maximum integration time step of 0.12 (instead of 0.01) leads to about 30 times faster C_10_E_4_ bilayer superstructure formation (with a simulation run below a single hour on a modest multi-core workstation) with a comparable result. The common “DPD best practice” of using a maximum integration time step of 0.04 still applies, as temperature drift becomes unphysical at larger time steps, but for an initial quick overview larger time steps can be tried to obtain successful hints, showing the potential of the DPD method for fast simulations with specific simulation setups.

A change of the “range 20” to the “range 15” particle set does not significantly alter the simulation results whereas the “range 10” particle set does no longer lead to the expected bilayer superstructure formation for C_10_E_4_ fragmentation scheme A. On the other hand, the “range 10” particle set still allows the formation of stacked bilayers for C_10_E_4_ fragmentation scheme B, corresponding to those of the “range 15” and the “range 20” particle set. Thus, an intrinsic interplay between molecule fragmentation and repulsion parameter scaling may be deduced, where the latter seems to have ranges of relative insensitivity but apparent lower thresholds beyond which the simulation approach fails. As a rule of thumb, a reasonable maximum scaling of the repulsion parameters seems to be an adequate as well as intuitive choice for a maximum discriminative behavior.

The overall performance of the different integration schemes suggests the S1MVV integrator to be a fast and good choice. This integrator was already highlighted in [21] as the “brightest star” at that time. In contrast, the later “outperforming” [22] PNHLN integrator may lead to the most convincing results for small integration time steps but exhibits unphysical artefacts for larger time steps which would be most important for its computational efficiency. In addition, the necessary empirical coupling parameter determination reduces its attractivity for practical application.

The findings for C_10_E_4_ fragmentation scheme B demonstrate the principal problem of adequate molecular fragmentation: Whereas scheme B still exhibits a successful C_10_E_4_ successful bilayer formation—in combination with being less sensitive to repulsion parameter scaling—the resulting bilayer thickness is less accurate compared to scheme A, thus scheme A may be regarded to be superior. Proper molecular decomposition into adequate particle structures remains to be a contentious issue where different approaches may finally be most suitable for reflecting different aspects of the system under study.

Volume scaling for concentration to molecule number mapping is advised by the findings of this work: A neglect of volume scaling would lead to an unrealistic shift towards higher C_10_E_4_ mass fractions that would less adequately reflect the experimental isotherm of the phase diagram.

Repulsion parameter morphing allows for an assessment of the sensitivity of the mutual repulsion parameter interplay to properly describe the molecular system under study. Since there is no general derivation scheme for repulsion parameter estimation, specific strategies for this task are commonly disputed in a controversial manner, e.g. concerning specific molecular mechanics force fields or specific water models as an adequate base for derivation procedures. The results for particle morphing presented in this work suggest that there is a considerable range of insensitivity that still allows for a proper mesoscopic description. Thus, detailed repulsion parameter accuracy issues should not be overstretched.

## Data Availability

Subfolder *2022 C10E4-water bilayer formation study* of MFsim repository at https://github.com/zielesny/MFsim
